# Capturing the Developmental Changes in Cognitive Control Engagement in Chinese Preschoolers

**DOI:** 10.3390/bs15020142

**Published:** 2025-01-28

**Authors:** Xufeng Ji, Yihao Deng, Qiong Zhang, Yanlin Zhou

**Affiliations:** 1 Faculty of Teacher Education, Lishui University, Lishui 323000, China; jixufeng@lsu.edu.cn; 2 Department of Psychology and Behavioral Sciences, Zhejiang University, Hangzhou 310058, China; dyhh217@zju.edu.cn (Y.D.); zhangqiongzgh@zju.edu.cn (Q.Z.)

**Keywords:** reactive control, proactive control, AX continuous performance task, cued task-switching paradigm

## Abstract

Young children typically engage in cognitive control reactively in response to specific situations, rather than proactively preparing for them. The developmental change from reactive to proactive control seems to happen gradually across early development and ultimately results in a qualitatively different behavior pattern. However, existing evidence is mainly based on cross-sectional designs. Thus, this study adopted a longitudinal design to examine the transition from reactive control to proactive control in preschoolers. Sixty preschoolers aged 4 (*n* = 31) and 5 (*n* = 29) were recruited and required to complete two cognitive control tasks (i.e., an AX-Continuous Performance Test and a Cued Task-Switching task) twice within a five-month interval. The results showed that the children improved their cognitive control skills across both tasks, demonstrating a predominantly reactive control pattern during the time interval. This improvement reflects an age-related gradual change, which is a preparation for evolving into a qualitatively different behavioral pattern over time. These findings provide longitudinal evidence for the developmental change from reactive to proactive control in early childhood strategies.

## 1. Introduction

Cognitive control generally refers to individuals’ ability to regulate, coordinate, and guide their thoughts and behaviors toward specific goals (Braver, 2012). These abilities are reported as predictors of academic achievement and success in other life domains (Moffitt et al., 2011). According to the Dual Mechanisms of Control framework, cognitive control abilities consist of two complementary modes: reactive control and proactive control (Braver et al., 2007; Braver, 2012). Reactive control can be described as a “late correction” mechanism that is recruited on an as-needed basis, such as when interference occurs (Braver, 2012). It is predominantly influenced by bottom–up inputs, driving the transient activation of the lateral prefrontal cortex, in combination with more widespread brain networks that include motor circuits (Ryman et al., 2019). In contrast, proactive control occurs through active maintenance of goal-relevant information, where interference can be dealt with through early-selection, top–down processes (Braver et al., 2009). It relies upon sustained activation of the lateral prefrontal cortex; therefore, it is metabolically more demanding than reactive control (Braver, 2012; Ryman et al., 2019). Despite these differences, it is critical for individuals to adaptively engage both reactive and proactive control in concert to maximize goal achievement.

As children grow up, they are increasingly able to employ efficient cognitive control processes, such as getting progressively more adept at organizing their actions, inhibiting inappropriate responses, and regulating their behaviors (Chatham et al., 2009; Chevalier et al., 2020; Gonthier et al., 2019). In addition, with increasing age, children can expand their repertoire of available strategies to address situational demands flexibly. Thus, those developmental changes in cognitive control are reflected by quantitative changes in terms of more efficient use of existing strategies and qualitative changes reflected by the implementation of new strategies (Chevalier, 2015; Gonthier et al., 2019).

When exploring these changes in strategy-employing, classical paradigms, including the AX-Continuous Performance Test (AX-CPT) and the Cued Task-Switching Paradigm (CTS), were popularly used. The AX-CPT mobilizes reactive and proactive control engagement through response inhibition (Kubota et al., 2020). In this task, participants are presented with a series of cue and target letters (i.e., an A cue or B cue followed by an X or Y probe) and are only required to respond positively to the AX sequence. Critically, AX trials occur frequently while the other trial types (AY, BX, and BY) rarely appear, creating a bias to select the target response when either the A cue or X probe appears. In this task, proactive control refers to the control engaged by the cue, whereas reactive control refers to the control driven by the probe. Thus, individuals who rely more on reactive control strategies will perform better on AY trials as compared to BX trials, while individuals who rely more on proactive control strategies will typically perform better on BX trials as compared to AY trials. Typically, young adults flexibly select the most adaptive control strategy in the AX-CPT (Braver et al., 2009; Iselin & DeCoster, 2009), which is reflected by better performance on BX than AY trails. In contrast, children show the aforementioned developmental changes from performing better on AY trials to doing better on BX trials, illustrating an age-related shift in control strategy (Chatham et al., 2009). Researchers have found that around the age of five, there is an emergence of a more prevalent proactive control strategy (Gonthier et al., 2019; Kubota et al., 2020; Lucenet & Blaye, 2014).

Using a CTS paradigm, Chevalier et al. (2015) tested children’s ability to engage in reactive or proactive control in the context of switching between the color-matching and shape-matching tasks. In both conditions, a task cue is displayed to signal the upcoming target (e.g., a palette of colors for color matching or a palette of geometric shapes for shape matching). An early cue presentation allows participants to deploy proactive control, including maintaining the task set and monitoring the upcoming task (Czernochowski, 2015; Blackwell & Munakata, 2014). Chevalier et al. (2015) manipulated the cue presentation into three conditions with different demands of cognitive control strategies. Specifically, in the Proactive-Impossible condition, the cue appeared simultaneously with the target, making it unnecessary to prepare in advance, thus prompting reactive control instead of proactive preparation. In the Proactive-Possible condition, the task cue was presented before the target and remained visible when the target appeared, making proactive preparation possible but unnecessary. Finally, in the Proactive-Encouraged condition, the cue was presented before the target but disappeared before the target’s appearance, incentivizing children to proactively attend to and process the cue. The authors reported that five-year-olds only engaged in proactive control strategies when specifically encouraged, while ten-year-olds used proactive control strategies whenever possible (Chevalier et al., 2015).

Those studies indicate a developmental change from reactive control to proactive control after the age of five years in both tasks (e.g., Blackwell & Munakata, 2014; Chevalier, 2015; Chevalier & Blaye, 2016; Doebel et al., 2017; Gonthier et al., 2019; Lucenet & Blaye, 2014; Niebaum & Munakata, 2020). However, a common limitation of previous studies is that they mostly rely on cross-sectional data and the use of one single task in isolation and, as such, provide an incomplete account of the developmental change of interest. Thus, the present study aimed to delineate the trajectory of reliance on reactive or proactive control in preschoolers by tracking the consecutive developmental changes after age 4 and using both the AX-CPT and CTS paradigms. We expected that compared to younger children, older children would be capable of deploying a strategy of proactive control, reflected by better performance on more proactive-demanding conditions (i.e., BX trials vs. AY trials in AX-CPT and *Proactive-Encouraged* trials in CTS).

## 2. Methods

### 2.1. Participants and Procedure

The sample size was determined through a priori power analyses based on previous evidence on the differences in proactive control between children aged five and six (Doebel et al., 2017; Gonthier et al., 2019; Kubota et al., 2020). Setting α at 0.05 and power (1-β) at 0.80; expecting a medium-to-large effect size of difference (Cohen’s *d* = 0.2), high correlations among repeated measures (*r* = 0.5), and nonsphericity correction = 1; and considering two age groups and two measurements, the power analysis (G*Power 3.1.9.4) indicated that a total sample size of at least 52 participants would yield adequate power to detect the changes in using a proactive control strategy.

We recruited two cohorts of typically developing Chinese preschoolers at a slightly younger age than in previous studies from two kindergartens that mainly served upper-middle-income families in Hangzhou, a city in southeastern China. Multiple pieces of evidence have shown that East Asian young children outperformed their Western counterparts on executive function tasks (e.g., Imada et al., 2013; Oh & Lewis, 2008; Sabbagh et al., 2006). It is possible that Chinese children would exhibit a different developmental picture in employing the cognitive control strategy (e.g., Imada et al., 2013; Oh & Lewis, 2008; Sabbagh et al., 2006). Children aged from 4 to 5 were recruited. The final sample consisted of thirty-one 4-year-olds (M_age_ = 4.20 years, range = 3.93−4.45 years; 48% girls) and twenty-nine 5-year-olds (M_age_ = 4.90 years, range = 4.79−5.02 years; 55% girls). All participants completed two performance-based assessments of cognitive control at two test sessions: initial recruitment (T1) and roughly five months later (T2). The test sequence across participants within each test session was counterbalanced; test sequence within participants across the test sessions was counterbalanced as well. Due to the unexpected outbreak of the Coronavirus Pandemic in 2019, we were not able to collect the data for a third timepoint, as initially planned. Our tasks were programmed in E-Prime 2.0, and children were tested on a laptop with a 14-inch display in a one-on-one setting in a quiet room during the school days. The ethical committee at our university approved this study. Written consent from caregivers and children’s verbal assent were obtained for all participants.

### 2.2. Measurements

AX-Continuous Performance Task (AX-CPT; Braver et al., 2005). This task presented participants with sequences of pictures, including pairs of cues (A = puppy, B = one of eight other animals) and probes (X = bone and Y = one of eight other types of food but not dog food, such as an apple or cabbage). Children were told a cover story that the puppy likes bones and that they should try to help the puppy (A) collect more bones (X). To do so, they were instructed to press a green button on the left when they saw a puppy followed by a bone (i.e., AX trials) and ignore the non-target animal–food pairs (i.e., AY, BX, and BY trials) by pressing a red button on the right. Every trial began with a cue presented centrally on the screen (500 ms), followed by a blank delay (1000 ms); then an X picture would display for 3000 ms, followed by another blank delay (300 ms). Children completed 16 practice trials to ensure task understanding. Ninety experimental trials were divided into three blocks, fifty-four of them (60%) being AX trials, and the remaining ones being AY, BX, and BY trials (12 trials of each type). The task lasted about 10 min. Accuracy and average response times (RTs; computed for correct responses only) were recorded separately for each of the four trial types (AX, AY, BX, and BY). The proactive behavioral index (PBI) was calculated as (AY − BX)/(AY + BX) separately for error rates (based on average error rates in AY and BX trials) and RTs (based on average RTs on AY and BX trials). The PBI reflects the relative balance of interference between AY and BX trials: a positive PBI reflects higher interference on AY trials, indicating proactive control, whereas a negative PBI reflects higher interference on BX trials, indicating reactive control (Braver, 2012; Chatham et al., 2009). Considering the trade-off between speed and accuracy, a composite PBI was also computed as the average (after standardization) of the error rate PBI and the response times PBI. Therefore, the composite PBI summarizes the use of proactive control with a single index, with positive values indicating more difficulty on AY trials than on BX trials (e.g., Gonthier et al., 2016, 2019).

Cued Task-Switching Paradigm (CTS, Chevalier et al., 2015). Children were required to sort toys (i.e., the targets) by either shape or color. On each trial, children first saw a fixation cross within a black circle for 1000 ms, followed by a gift box, which contained the toy that had to be sorted in the next step. A colored dot presented in the circle served as a cue and instructed children to sort targets by color; a gray triangle cue presented in the circle indicated them to sort targets by shape; and a brown circle instructed children to wait for the next stimuli to make a judgment. There were the following three cue conditions: First, in the “Proactive-Impossible” condition, non-informative brown circles were presented first, and the task cue was displayed simultaneously with the target, hence preventing proactive preparation and encouraging reactive control. Second, in the “Proactive-Possible” condition, the cue was presented along with the gift. It remained visible after target onset, allowing children to prepare proactively for the upcoming target. However, children could still reactively process the task cue after target onset, so cue-based proactive preparation was possible but unnecessary. Finally, in the “Proactive-Encouraged” condition, early cue presentation was terminated ahead of target presentation, making reactive control more challenging and encouraging children to memorize or process the cue proactively. Cue conditions were blocked, and the order was counterbalanced across participants. Participants were explicitly informed of the change in cue presentation as they started a new condition. Each condition started with two demonstration trials completed by the experimenter, followed by the child completing practice trials with feedback. In the test phase, children completed three blocks of 21 trials separated by short breaks. The task lasted about 16 min. Faster and more accurate responses on the Proactive-Possible and Proactive-Encouraged conditions compared to the Proactive-Impossible condition suggest the engagement of a proactive strategy when sorting with the indicated dimension. In contrast, slower and less accurate performance on the Proactive-Impossible and Proactive-Possible conditions compared to the Proactive-Encouraged condition indicates the engagement of a predominantly reactive strategy, i.e., the processing of the cue once the target appears. Moreover, significantly faster RTs in the Proactive-Encouraged condition as compared with the other two conditions indicate the ability to use proactive control when encouraged to do so (Chevalier et al., 2015; Doebel et al., 2017).

### 2.3. Data Pre-Processing

In the AX-CPT, the first trial in each block (always an AX trial due to the pseudo-random order) was excluded from the analyses. Trials with response times below 200 ms and trials with no responses (less than 3% of the data) were also excluded. In the CTS, trials with response times below 200 ms (less than 3% of the data) were excluded.

## 3. Data Analysis and Results

Descriptive statistics, test–retest reliability, and within-group comparisons for both tasks, the AX-CPT and CTS, are presented in Table 1.

### 3.1. AX-CPT

**Figure 1:**
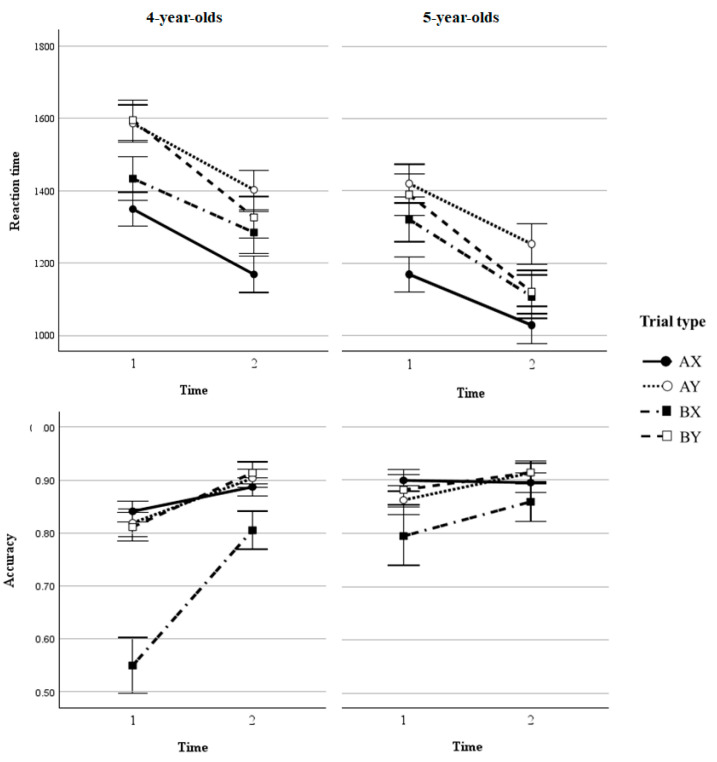
Mean reaction times (**top row**) and accuracy rates (**bottom row**) for 4-year-olds (**left**) and 5-year-olds (**right**) as a function of each trial type and test session in the AX-CPT. Error bars indicate standard errors of the mean.

We first ran three-way repeated-measures ANOVA tests to evaluate the effects of age group (4-year-olds/5-year-olds), test session (T1/T2), and trial type (AX/AY/BX/BY) on reaction time and accuracy (cf. Figure 1).

Reaction time. Our analyses revealed a significant main effect of the test session, indicating that all participants performed significantly faster at T2 compared to T1 (*F*(1, 174) = 29.44, *p* < 0.001, *η*^2^*_p_* = 0.19; cf. Table 2). The main effect of the trial type was also significant (*F*(3, 174) = 29.03, *p* < 0.001, *η*^2^*_p_* = 0.33); planned comparisons showed that the reaction times in BX trials (1283.67 ms) were significantly shorter than those in AY trials (1415.43 ms) (*p* < 0.001, *η*^2^*_p_* = −0.62). A significant main effect of the age group indicated that 5-year-olds responded faster than 4-year-olds (*F*(1, 58) = 9.12, *p* = 0.004, *η*^2^*_p_* = 0.14). There were no significant two-way interactions between test session and age, trial type and age, or test session and trial type (all *p*s > 0.05). The three-way interaction was not significant either (*p* > 0.05).

Accuracy rate. The main effect of the test session on accuracy was significant (*F*(1, 174) = 27.82, *p* < 0.001, *η*^2^*_p_* = 0.32), indicating that all participants performed more accurately at T2 compared to T1. The main effect of the trial type was also significant (*F*(3, 174) = 17.31, *p* < 0.001, *η*^2^*_p_* = 0.23); planned comparisons showed that the accuracy of BX trials (0.75) was significantly lower than that of AY trials (0.87, *p* < 0.001). A significant main effect of the age group indicated that 5-year-olds responded more accurately than 4-year-olds (*F*(1, 58) = 7.25, *p* = 0.009, *η*^2^*_p_* = 0.11). The interaction between the test session and trial type was also significant (*F*(3, 174) = 7.06, *p* < 0.01, *η*^2^*_p_* = 0.11). Simple effect analyses showed that from T1 to T2, children displayed significant improvements in BX trials (0.67 vs. 0.83, *p* < 0.001) and AY trials (0.84 vs. 0.91, *p* = 0.001). In addition, children performed significantly better in AY trials than in BX trials at T1 (*p* < 0.001); this difference was observed at T2 as well (*p* = 0.008). The interaction between the test session and age was also significant (*F*(1, 174) = 8.23, *p* = 0.006, *η*^2^*_p_* = 0.13). Simple effect analyses showed that 4-year-olds’ performance at T1 (0.76) was significantly worse than their performance at T2 (0.88, *p* = 0.001), and 5-year-olds’ performance at T1 (0.86) was not significantly different from that at T2 (0.90, *p* = 0.101). The three-way interaction with the factors test session, trial type, and age group was also significant (*p* = 0.049, *η*^2^*_p_* = 0.04). We found that from T1 to T2, 4-year-olds evidently improved their accuracy in BX (*p* < 0.001) and AY trials (*p* < 0.01). Furthermore, they showed better accuracy in AY trials than in BX trials at both T1 (*p* < 0.001) and T2 (*p* = 0.013). For 5-year-olds, neither the accuracy of AY trials (*p* = 0.08) nor that of BX trials (*p* = 0.21) failed to improve significantly from T1 to T2. The difference in accuracy rate between AY and BX trials was not significant, either at T1 (*p* = 0.25) or T2 (*p* = 0.18). Taken together, these results suggest a significant performance improvement in BX trials from T1 to T2, but only for the 4-year-old group (cf. Figure 1).

Composite PBI. To investigate the developmental transition from reactive to proactive control, we ran two two-way ANOVA tests using age group (4-year-olds/5-year-olds) as a between-subjects factor and test session (T1/T2) as a within-subject factor using the composite PBI as outcome measures. The effect size estimates are provided as partial eta-square (*η*^2^*_p_*) for the ANOVA tests and as Cohen’s *d* for paired comparisons (Cumming, 2012).

**Figure 2:**
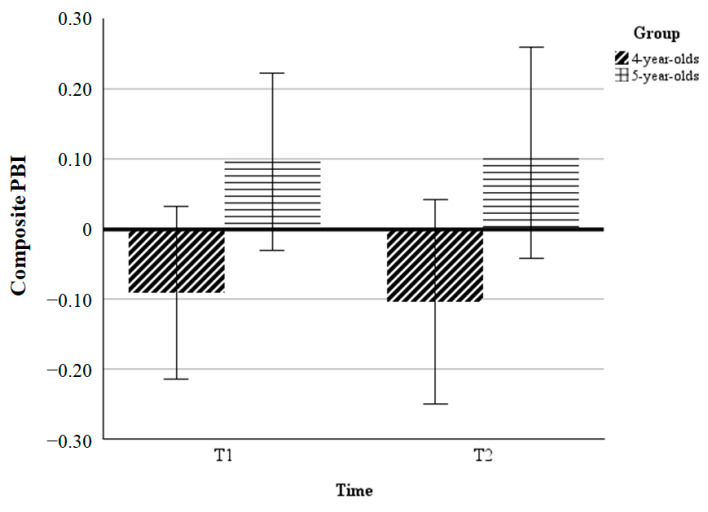
Mean composite PBI for 4-year-olds and 5-year-olds as a function of test session in the AX-CPT. Error bars indicate standard errors of the mean.

As shown in Figure 2, the results showed that the main effect of the test session (*F*(1, 58) = 0.00, *p* = 0.997, *η*^2^*_p_* = 0.00), the main effect of the age group (*F*(1, 58) = 1.60, *p* = 0.21, *η*^2^*_p_* = 0.03), as well as the age group by test session interaction were all not significant (*F*(1, 58) = 0.01, *p* = 0.91, *η*^2^*_p_* = 0.00).

### 3.2. CTS

**Figure 3:**
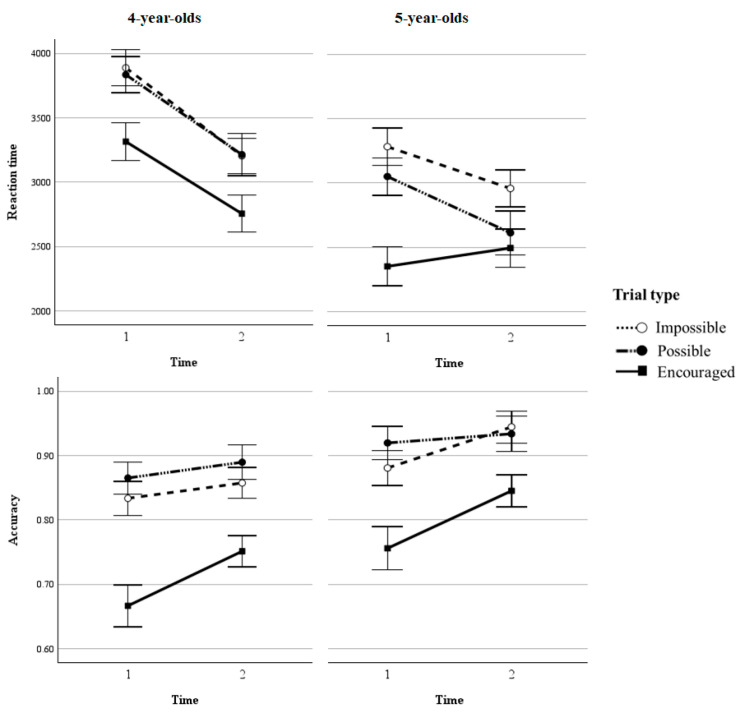
Mean reaction times (**top row**) and accuracy rates (**bottom row**) for 4-year-olds (**left**) and 5-year-olds (**right**) as a function of each trial type and test session in the Cued Task-Switching paradigm. Error bars indicate standard errors of the mean.

To analyze the CTS performance, we conducted similar 2 (age group: 4-year-olds/5-year-olds) × 2 (test session: T1/T2) × 3 (trial type: Proactive-Impossible/Possible/Encouraged) ANOVA tests for reaction time and accuracy (cf. Figure 3).

Reaction time. The significant main effect of the test session revealed a shorter reaction time at T2 than at T1 (*F*(1, 58) = 13.40, *p* < 0.01, *η*^2^_*p*_ = 0.19; cf. Table 2). The significant main effect of trial type (*F*(2, 116) = 30.18, *p* < 0.001, *η*^2^_*p*_ = 0.34) showed that the reaction times of Proactive-Encouraged trials were shorter than those of Proactive-Impossible trials (*p* < 0.001) and Proactive-Possible trials (*p* < 0.001, *d* = 0.72), indicating that proactive control strategy is beneficial for processing speed. A significant main effect of the age group (*F*(1, 58) = 20.55, *p* < 0.001, *η*^2^_*p*_ = 0.26) showed that 5-year-olds performed faster than 4-year-olds. The interaction between the test sessions and trial types was significant as well (*F*(2, 116) = 3.36, *p* = 0.038, *η*^2^_*p*_ = 0.06). Simple effect analyses showed that compared to T1, children’s performance at T2 was significantly faster in Proactive-Impossible trials (*p* < 0.001) and in Proactive-Possible trials (*p* < 0.001) but not in Proactive-Encouraged trials (*p* = 0.15). Reaction times of Proactive-Impossible trials were significantly longer than those of Proactive-Encouraged trials at both T1 (*p* < 0.001) and T2 (*p* < 0.001). The reaction times of Proactive-Possible trials were longer than those of Proactive-Encouraged trials, both at T1 (*p* < 0.001) and T2 (*p* < 0.01). Neither the two-way interaction between the trial type and age group, test session and age group, nor the three-way interaction was significant (*p*s > 0.05).

Accuracy rate. A significant main effect of the test session showed higher accuracy at T2 than at T1 (*F*(1, 58) = 8.71, *p* = 0.005, *η*^2^_*p*_ = 0.13). A significant main effect of the trial type (*F*(2, 116) = 45.35, *p* < 0.001, *η*^2^_*p*_ = 0.44) indicated that the accuracy of Proactive-Encouraged trials (0.76) was significantly lower than that of Proactive-Impossible trials (0.90, *p* < 0.001, *d* = −0.96) and Proactive-Possible trials (0.88, *p* < 0.001). A significant main effect of age showed that 5-year-olds performed more accurately than 4-year-olds (*F*(1, 58) = 8.58, *p* < 0.01, *η*^2^_*p*_ = 0.13). A significant interaction between the test session and trial type (*F*(2, 116) = 3.11, *p* = 0.048, *η*^2^_*p*_ = 0.05) showed that from T1 to T2, children’s performance improved significantly on Proactive-Encouraged trials (*p* < 0.001), but not on either Proactive-Impossible trials (*p* = 0.07) or Proactive-Possible trials (*p* = 0.41). The test session by age group interaction, the trial type by age group interaction, and the corresponding three-way interaction were all not significant. In conclusion, the results of CTS suggested that both age groups improved similarly from T1 to T2 (*p*s > 0.05, cf. Figure 3).

## 4. Discussion

An analysis of previous literature shows that preschoolers undergo a developmental change from relying on reactive control to implementing more proactive control strategies. However, the existing research has been limited in that most studies have been cross-sectional and/or relied on single outcome measures, and thus, longitudinal evidence with multiple testing is greatly required. To address this gap, we investigated the development in the implementation of cognitive control strategies using a longitudinal design. Using two groups of preschoolers (4-year-olds and 5-year-olds) who performed two cognitive control paradigms (i.e., AX-CPT and CTS) twice within a five-month interval, we found that children demonstrated a transition towards less reactive control strategy during this time window. However, we failed to elicit a significantly more proactive control strategy employment. In addition, insignificant correlation was found between the two measurements for cognitive control strategy mobilization.

Considering the performance in AX-CPT, the 4-year-olds showed a general improvement from T1 to T2. They demonstrated a predominantly reactive profile in this task at T1, with higher error rates in BX trials than in AY trials; this difference persisted at T2, but the overall performance improved. This finding indicates that 4-year-olds’ showed a shift towards less reactive control mobilization. Although 5-year-olds reacted dramatically faster at T2 than at T1, their accuracy on the four trial types remained stable, suggesting no significant change in strategy use at this age range. Moreover, an examination of the composite PBI provided further evidence of gradual changes in the use of cognitive control strategies. Although previous studies reported that there is a significant transition from a tendency to act reactively to proactively after 5, our work suggested that at least from 4.2 years to 5.4 years, the use of cognitive control strategy still undergoes a quantitative change (Andrews-Hanna et al., 2011; Chevalier et al., 2014, 2015; Gonthier et al., 2019; Lorsbach & Reimer, 2010; Lucenet & Blaye, 2014).

Similarly, the results of the CTS showed that from T1 to T2, the performance of all children is equivalent in the Proactive-Impossible and Proactive-Possible trials, indicating that none of them make use of proactive control when it is possible and that the transition towards proactive control has not occurred yet.

In conclusion, the results from both cognitive control tasks showed that young children progressively employ less reactive control strategies between the ages of four and five. The reason that we failed to find a qualitative shift might be due to a relatively earlier age range. This setting in participant recruiting was based on previous findings that Chinese preschoolers outperform their Western peers in various cognitive control tasks (e.g., Ng et al., 2015; Oh & Lewis, 2008; Sabbagh et al., 2006). As such, we assumed that Chinese children might display an earlier shift from reactive control to proactive control engagement compared to Western children; this was not confirmed, and it suggested that proactive control engagement and performance in cognitive tasks are not consistent, especially in childhood.

Additionally, although the performance in the two tasks is consistent, we failed to elicit a correlation between the two measurements, which partly echoes with what Kubota et al. (2020) reported, that is, cognitive control strategy engagement across contexts might be influenced by other cognitive abilities. As children are experiencing vast development in diverse abilities in preschool years, a cross-task association might be relatively low. In addition, the requirements for the two tasks are different. That is, AX-CPT might roughly capture the reliance on a balance between the reactive control or proactive control strategy recruitment, which fails to indicate whether and how children are on the way to shifting into the flexible use of proactive strategies (Doebel et al., 2017). By manipulating the timing of the cue presentation in the Proactive-Impossible/Possible/Encouraged conditions, the CTS allows us to obtain more specific and detailed information on whether a child can spontaneously use a proactive control strategy or not (e.g., Chevalier et al., 2015; Czernochowski, 2015). As the majority of previous studies investigating the developmental change in cognitive control during early childhood have relied exclusively on the AX-CPT (e.g., Chatham et al., 2009; Gonthier et al., 2019; Lucenet & Blaye, 2014), we provided further evidence by multi-task facets. Although the performance in both tasks suggested a quantitative change of strategy-using at this particular age range in our study, more multiple testing is suggested to uncover the developmental change of cognitive control strategy employment.

Nevertheless, our study has some limitations that motivate further research. Firstly, we used the same cognitive tasks across two test sessions, making it hard to disentangle general practice and learning effects from maturational changes. However, different improvement patterns for the two age groups might minimize the possibility of a general practice effect. Secondly, due to the unexpected outbreak of the Coronavirus Disease in 2019, we were restricted from collecting data for a third timepoint, which would have allowed us to draw firmer conclusions about the developmental trajectories of reactive and proactive control. Another caveat is that our population consisted of typically developing children from predominantly privileged backgrounds, and thus, our results might have been affected by restricted range issues. Overall, future studies would benefit from adopting longitudinal designs across multiple time points and recruiting a larger sample that includes children from more diverse backgrounds.

## 5. Conclusions

This study employed a longitudinal design to simultaneously track 4-year-old and 5-year-old children’s developmental change in reactive control and proactive control using two cognitive control tasks (AX-CPT and CTS). Children predominantly responded in a reactive pattern after a 5-month interval. This improvement reflects an age-related gradual change, which is a preparation for evolving into a qualitatively different behavioral pattern over time.

## Figures and Tables

**Table 1:** Descriptive statistics and within-group comparisons of the AX-Continuous Performance Test (AX-CPT) and the Cued Task-Switching Paradigm (CTS) at both test occasions.

		Reaction Time (ms)						Accuracy					
		T1		T2		T1 vs. T2		T1		T2		T1 vs. T2	
	Trial Type	*M*	*SD*	*M*	*SD*	*t*	*d*	*M*	*SD*	*M*	*SD*	*t*	*d*
AX-CPT	4-year-olds (*n* = 31)												
	AX	1350	274	1169	327	2.90 **	0.52	0.84	0.12	0.89	0.10	−1.67	−0.30
	AY	1586	281	1402	336	2.89 **	0.52	0.82	0.15	0.90	0.11	−3.00 **	−0.54
	BX	1434	344	1285	327	2.43 *	0.44	0.55	0.33	0.81	0.22	−4.66 ***	−0.84
	BY	1596	317	1327	322	4.36 ***	0.78	0.81	0.16	0.91	0.11	−3.20 **	−0.58
	PBI	0.06	0.12	0.05	0.13	0.32	0.08	−0.30	0.45	−0.18	0.44	−1.19	0.27
	5-year-olds (*n* = 29)												
	AX	1169	241	1028	218	3.00 **	0.56	0.90	0.09	0.90	0.10	0.29	0.05
	AY	1420	298	1253	266	2.85 **	0.53	0.86	0.15	0.91	0.09	−1.85	−0.34
	BX	1321	323	1107	321	2.67 *	0.50	0.80	0.25	0.86	0.17	−1.47	−0.27
	BY	1390	305	1120	325	3.90 ***	0.72	0.88	0.15	0.91	0.13	−1.19	−0.22
	PBI	0.14	0.10	0.07	0.11	−1.11	0.67	−0.06	0.47	−0.07	0.48	0.15	0.02
CTS	4-year-olds (*n* = 31)												
	Impossible	3889	854	3204	699	3.27 **	0.59	0.83	0.14	0.86	0.17	−0.64	−0.12
	Possible	3835	765	3214	869	3.08 **	0.55	0.87	0.16	0.89	0.16	−0.76	−0.14
	Encouraged	3315	982	2755	664	2.83 **	0.51	0.67	0.18	0.75	0.14	−2.39 *	−0.43
	5-year-olds (*n* = 29)												
	Impossible	3282	708	2957	842	1.94	0.36	0.88	0.15	0.94	0.07	−2.28 *	−0.42
	Possible	3050	802	2612	960	2.47 *	0.46	0.92	0.12	0.93	0.13	−0.42	−0.08
	Encouraged	2351	580	2494	920	−0.67	−0.13	0.76	0.18	0.85	0.13	−3.23 **	−0.60

Note: 4-year-olds (*n* = 31); 5-year-olds (*n* = 29); T1 = test session 1; T2 = test session 2; PBI = proactive behavioral index. * *p* < 0.05, ** *p* < 0.01, *** *p* < 0.001.

**Table 2:** Three-way repeated-measures ANOVA of mean reaction time and accuracy rate in the AX-Continuous Performance Task (AX-CPT) and the Cued Task-Switching paradigm (CTS).

	AX-CPT						CTS					
	Reaction Time			Accuracy			Reaction Time			Accuracy		
	F	*p*	*η* ^2^ *_p_*	F	*p*	*η* ^2^ *_p_*	F	*p*	*η* ^2^ *_p_*	F	*p*	*η* ^2^ *_p_*
Test session	29.44	<0.001 ***	0.34	27.82	<0.001 ***	0.32	13.40	<0.001 ***	0.19	8.71	0.005 **	0.13
Trial type	29.03	<0.001 ***	0.33	17.31	<0.001 ***	0.23	30.18	<0.001 ***	0.34	45.35	<0.001 ***	0.44
Age group	9.12	0.004 **	0.14	7.25	0.009 **	0.11	20.55	<0.001 ***	0.26	8.58	0.005 **	0.13
Test session × Trial type	2.57	0.056 ^+^	0.04	7.06	<0.001 ***	0.11	3.36	0.038 *	0.06	3.12	0.048 *	0.05
Test session × Age group	0.00	0.977	0.00	8.29	0.006 **	0.13	3.37	0.07 ^+^	0.06	0.11	0.740	0.00
Trial type × Age group	0.50	0.685	0.01	3.79	0.011 *	0.06	1.43	0.243	0.02	0.82	0.445	0.01
Test session × Trial type × Age group	0.53	0.660	0.01	2.67	0.049 *	0.04	1.85	0.163	0.03	0.44	0.645	0.01

Note: ^+^ *p* < 0.1, * *p* < 0.05, ** *p* < 0.01, *** *p* < 0.001.

## Data Availability

The raw data supporting the conclusions of this article will be made available by the authors on request.
